# Bio-Ethics and One Health: A Case Study Approach to Building Reflexive Governance

**DOI:** 10.3389/fpubh.2022.648593

**Published:** 2022-03-18

**Authors:** Antoine Boudreau LeBlanc, Bryn Williams-Jones, Cécile Aenishaenslin

**Affiliations:** ^1^Bioethics Programs, Department of Social and Preventive Medicine, Public Health School, Université de Montréal, Montréal, QC, Canada; ^2^Department of Pathology and Microbiology, Faculty of Veterinary Medicine, Université de Montréal, Montréal, QC, Canada

**Keywords:** pragmatic ethics, program governance, data access, One Health surveillance, responsibility, antimicrobial use (AMU), sustainable development

## Abstract

Surveillance programs supporting the management of One Health issues such as antibiotic resistance are complex systems in themselves. Designing ethical surveillance systems is thus a complex task (retroactive and iterative), yet one that is also complicated to implement and evaluate (e.g., sharing, collaboration, and governance). The governance of health surveillance requires attention to ethical concerns about data and knowledge (e.g., performance, trust, accountability, and transparency) and empowerment ethics, also referred to as a form of responsible self-governance. Ethics in reflexive governance operates as a systematic critical-thinking procedure that aims to define its value: What are the “right” criteria to justify how to govern “good” actions for a “better” future? The objective is to lay the foundations for a methodological framework in empirical bioethics, the rudiments of which have been applied to a case study to building reflexive governance in One Health. This ongoing critical thinking process involves “mapping, framing, and shaping” the dynamics of interests and perspectives that could jeopardize a “better” future. This paper proposes to hybridize methods to combine insights from collective deliberation and expert evaluation through a reflexive governance functioning as a community-based action-ethics methodology. The intention is to empower individuals and associations in a dialogue with society, which operation is carried out using a case study approach on data sharing systems. We based our reasoning on a feasibility study conducted in Québec, Canada (2018–2021), envisioning an antibiotic use surveillance program in animal health for 2023. Using the adaptive cycle and governance techniques and perspectives, we synthesize an alternative governance model rooted in the value of empowerment. The framework, depicted as a new “research and design (R&D)” practice, is linking operation and innovation by bridging the gap between Reflexive, Evaluative, and Deliberative reasonings and by intellectualizing the management of democratizing critical thinking locally (collective ethics) by recognizing its context (social ethics). Drawing on the literature in One Health and sustainable development studies, this article describes how a communitarian and pragmatic approach can broaden the vision of feasibility studies to ease collaboration through public-private-academic partnerships. The result is a process that “reassembles” the One Health paradigm under the perspective of global bioethics to create bridges between the person and the ecosystem through pragmatic ethics.

## Introduction

The greatest health, social, and environmental challenges of the twenty-first century, such as antibiotic resistance, zoonotic pandemics, and climate change, require a “complexification” of monitoring and management programs (1). One Health aiming at the convergence of human, animal, and environmental health seeks to operationalize this complexity—in terms of contextualization, participation, and adaptation—through the integration of adaptive governance systems in evaluation, surveillance, and intervention (2–4). However, such programs should also be based on the practice of empowerment ethics: a self-critical examination, a receptivity to criticism, and a critical duty to change in order to judge and implement “good” learning for a “better future.” As understood by Van Rensselaer Potter (1911–2001; who coined the term bioethics in 1970), empowerment must lead to responsibility as a duty, not only to autonomy as a right for self-governance which tends to separate the singular will from biological facts (5, 6). First, em-power (*in/within* power) means managing and preventing the power, knowledge, and interest dynamic that transcends and modulates people and “community-will” and behavior: a “bottom-up” approach. Second, empowerment must lead individuals and communities to make their own changes, as autonomous and self-determining agents who acknowledge local values and constraints as criteria for change. Third, power must lead to awareness of our individual and social actions, which should also lead to self-responsibility and even to accountability mechanisms: an “abductive” approach (7)^1^.

Data sharing processes, biosurveillance programs, and multiscale analysis are techno-intellectual systems; they are examples of Edgar Morin's complexity concept. Managing complex systems requires pragmatic methodologies and ethics (9) because the willingness of people to consent to and participate in these systems changes over time (10, 11). An ethic (as a code) is useful both for transparency and as a means to produce a climate of trust that is suitable for discussion, while ethical analysis (as a methodology) is a key component to avoid the emergence of oppressive powers, to reduce bias, and to envision a “better future” (12, 13). For instance, feedback from One Health surveillance programs such as benchmarking is appreciated by practitioners where it improves their practices, until it raises ethical issues, notably, risks of bias and confidentiality breach. Empowerment ethics (as a discipline), then, is about finding ways to communicate to produce a collective narrative that gives meaning and orientation to actions and is especially important when there is a diversity of terminologies and interdisciplinary perspectives. In this view, communication must go beyond the person-to-person exchange and become a collective process (14).

Predictive and mechanistic models are no longer sufficient to address health problems in all their complexity (15). Callon et al. (14) propose that we move from risk assessment and management to a social process that integrates more broadly uncertainty. One Health should therefore move its problem-solving strategies “upstream,” before program ideation, which we will call here the “assemblage” of its knowledge, notably, its technologies, its methods, even its paradigms (16, 17). Such One Health ethics, which would progress through communication and knowledge systems, would enable stakeholders to question and advance their understanding and positioning. However, managing the “cross” thematic (e.g., human, animal vs. environment studies), the “inter” disciplines (e.g., medicine, technology, and law), the “trans” sectorial (e.g., the relationship between experts and non-specialists), and the “multi” scale viewpoints (e.g., human, beings, and things as organizational units) is a fundamental barrier to the successful implementation of One Health programs (18–20).

The objective is to lay the foundations for a methodological framework in empirical bioethics, the rudiments of which have been applied to a case study to building reflexive governance in One Health. This case made it possible to study the functioning of empowerment ethics in the development cycles of One Health surveillance programs. The proposed case sought to implement an antibiotic use surveillance program in animal health (2018–2021) in Quebec, Canada. Data from this surveillance system in public health could allow both scientific research and informed decision-making. The research question here, in bioethics, is about the promotion and education of critical thinking in technology and health, not only of scientists and policymakers but of all stakeholders. Building on a pragmatic approach to bioethics, this reasoning is driven by, first: How could empowerment ethics ease the “bridging” between cognitive and collective? From a synthesis of One Health methodologies and paradigms, the reasoning continues with a framework laying out the theoretical rudiments for a hybrid method to understand: How can we embed person-to-person dialogue in a collective assemblage to engage social groups in a negotiation process? Dialogue is supported by reflective critical thinking approaches, while social negotiation moves this cognitive to the level of collective interaction supported by ethical deliberation approaches. Finally, learnings from the case study will give answers to How can a community of beings and things become “reflexive,” conscious, and responsible, i.e., the “ought to be” empowered?

## Site and Approach

### Case Study of Antimicrobial Surveillance and Data Governance

Since 2017, the Government of Quebec has been considering developing surveillance programs that integrate efforts in health prevention between several ministries and sectors (see the Government Policy on Health Prevention; GPHP). This type of policy could be improved by its connection with several advanced theories in various fields that converge in practice under the perspective and terminologies of One Health and sustainable development. A challenge for One Health, in such a policy context, is to deepen the decision-maker's understanding of the pertinent theories while avoiding reduction to a set of expert buzzwords or jargon that then complicate translation into practical terms (21).

One of the policy objectives set by the Government was to develop a well-articulated program that had community-based meaning and criteria (e.g., feasible and acceptable, understood as useful, and sustainable) for a surveillance system of antibiotic use in animal health (agriculture and pets). Its program arose in a social context where several sectors, particularly in human, animal, and environmental health, had already implemented initiatives dealing with outbreaks, pharmaceuticals, and, notably, antimicrobial governance based on systems of information and communication technologies (SICT) used for surveillance purposes. In animal health, different committees, associations, and groups are involved in leading these reflections through participation in formal and informal working groups. These activities aim to produce detailed application procedures that would then be laid out in an action plan for the management of antibiotic use, surveillance, and governance for all sectors of veterinary practice and food production^2^. Many initiatives were more local (microscale) and launched by individuals or groups affiliated with the industry, academia, and professional associations, while others were broader (macroscale), and initiated by governments and civil society as networks (market, culture, values, etc.). The resulting initiatives are designed to push for change on both a micro and macro scale. But for such change to be effective, it requires a mixture of approaches, including both “top-down” political incentives (regulations, financing, and infrastructure) and “bottom-up” processes, including democratic mechanisms.

In light of these different initiatives, the *Ministère de l'agriculture, des pêcheries et de l'alimentation* (MAPAQ) mandated a feasibility study of the implementation of a surveillance program on the use of antibiotics in animals in Quebec (2018–2021). Several recommendations emerged from the resulting consensus-building process (2019–2020) within the veterinary and agricultural community, notably:

1. To build data systems and information platforms and their use based on trust,
2. To co-construct a common normative language,
3. To design a collaborative governance regime to shape the functioning of the program.

Aiming to implement a methodology for this collaborative collective thinking, the multidisciplinary team in charge of the feasibility study made a distinction between “consultation” and “concertation” to unpack the consensus-building nature of the methodological process. The consultation aims to gather information from a group to inform experts in developing the “best” model possible (the feasibility aspect), while concertation seeks for consensus among a group to deliberate about which criteria are “best” (values and vision), in order to give an acceptable orientation to the developing model (see here the clear distinction between descriptive and appreciative knowledge further developed in the last section).

The concertation phase of the feasibility study, which began with 60 representatives, eventually brought together 100, an extensive recruitment process aimed at saturating the perspectives covered by the different sectors of activity (industry, academia, government, association, order, etc.), practice (pork, poultry, small and large ruminants, pets, and sports animals), and professions (breeders, veterinarians, nutritionists, researchers, informatics experts, etc.). The two concertation events made it possible to collectively deliberate on the overall vision and were then followed by 12 consultations that brought together different stakeholders in small groups (6–16 participants per group) to deepen the discussion. Subsequent focused individual interviews made it possible to add reflexive details to the perspectives (challenges and facilitators) of key actors involved in the process (e.g., data, software, IT support providers). Human and environmental sectors (professional, academic, political, and industrial) were not the focus of the discussions; instead, the focus was on the ethical challenge of implementing a new technosocial program and developing a policy to manage the use of antibiotics in animal health. Nevertheless, many of the participants were invited to group discussions and interviewed separately to further explore their views on how the animal health situation is nested within the larger context of One Health. Ethics approval was received for the research phase of this project from the University Research Ethics Committee (anonymous); all participants were informed of the nature and scope of the project, the confidentiality mechanisms in place, and gave their consent to participate.

### A Pragmatic Bioethics Approach

One of the notable challenges of contemporary methodologies is to account for multi-scale relationships, building a bridge between the individual, the social, and the global. Ten Have (22) introduces the community perspective as an appropriate approach to operating global bioethics. By examining recent conceptual advances in pragmatic ethics and empirical bioethics, we propose here the operation of a community-based action-research (translated in action-ethics). According to Jonathan Ives (23) synthesis of methodologies in empirical bioethics (24, 25), an ethical analysis of a complex situation should be reflexive and focus on the empirical case under study, and not be biased by it, and so risk (over)valuing preexisting injustices rather than criticizing them (26). A multidisciplinary team of academics (veterinary medicine, bioethics, and law) and practitioners (veterinary, farmers, millers, association, and industry) contributed extensively to the empirical bioethics research project, which was embedded within and thus part of the above-mentioned feasibility study.

The team located at the *Faculté de Médecine Vétérinaire* at the *Université de Montréal* (FMVUM) was mandated by MAPAQ to conduct a large-scale empirical and social project. By reviewing, commenting upon, and critiquing the rudiments of this philosophical model, both the team and participants contributed to testing and enriching the model, as intended by *good* reflective practice. As an employee for this project, one of the authors, Boudreau LeBlanc (a Ph.D. student and empirical bioethicist in training), was given the task to develop a model to manage the ethical challenges with deploying consultation and concertation processes and to ensure the reflexivity of the experts and the deliberation of the collective (e.g., power relations, naturalistic, and philosophical reasoning biases). One solution to the challenge of bridging reflexivity and deliberation was to include the micro, meso, and macroscales in discussions, although this also introduced other ethical challenges, namely the subjectivity of each actor (e.g., their values, knowledge, and interests). Thus, it was obvious that a collective ethic was needed to set ground rules so that the actors could cooperate and co-construct the governance system. The feasibility study (2018–2021) conducted in Quebec, Canada, to evaluate the possible implementation of a monitoring system for the use of antibiotics in veterinary medicine was thus an ideal opportunity to study *in situ* how such a collective ethic could be implemented in a complex system in a way that leads to empowerment (27, 28).

Ethics is crucial to guiding new means of collaboration through public-private-academic partnerships, but it also requires empirical methodologies such as those developed in bioethics and pragmatism. Pragmatic ethics is about negotiating conflicting positions that emerge from empirical situations (within the community) and are made accessible through philosophical reasoning (26, 29). Ethics of science means (here, as a discipline) supporting the scientific community to question itself, individually as the responsible conduct of researchers, but also collectively through critiques of the nature of scientific research (in general and related to specific projects). Ethics (as in codes) is involved in the development of appreciative criteria and knowledge to judge conduct, evaluate the purpose, and propose appropriate governance mechanisms. Such an understanding of ethics could favor trust and lead to an agreement between experts and the community, which could then accelerate change. However, any criteria also need to progress through time *via* both rational reflexivity (a cognitive process) and collective deliberation (a social process). Without pragmatism in the ethics of science, researchers and communities will struggle to produce effective and adaptive networks, norms, and actions because the interests of the various stakeholders (as social or disciplinary) will not be articulated or aligned (5, 6).

A practice of empowerment ethics should be developed by and for a community (30, 31). In this Potterian One Health framing, empowerment ethics is presented as an alternative mode of governance that returns power to the key stakeholders involved, so that they can become actors of and responsible for the changes with which they will live (6). Such an understanding of ethics could provide a valuable foundation on which to build collaborative governance regimes that transcend academic, industrial, and public service limitations (32, 33). One of the cores aims of empowerment ethics is to build and maintain the trust of the key stakeholders involved in the program (34, 35). According to Davet et al. (36), three methodological processes should be used:

1. Design a practical model of change,
2. Set up a collaborative governance structure early in the thinking process,
3. Co-construct a common language to give purpose to, and a framework for, points 1 and 2.

As theorized by Latour (16), such a method necessitates a socio-episteme-methodological approach, the sort which is embodied in One Health and, more broadly, in bioethics (37). In practice, such an approach needs to be distributed at various scales of observation, at all levels of knowledge, and across several analytical dimensions, explained by Callon et al. (14) as three intertwined operations of translation and by Latour (16) as the metrological manner of constructing scientific standards (see the synthesis tool in the last section).

This ethics-science-society organizational approach highlighted in Beever and Whitehouse's (37) work in Potterian bioethics is conceptually close enough to Morin's philosophy to be qualified as a “Penser Global” (“Think Global,” not “worldwide,” 2015) or more deeply to fall under the paradigm of human complexity (38), and requires an extended understanding of the sciences and an answer to the question: what is “truly” feasible? In a similar vein, the UN (39) has called for a science of sciences to develop an ecosystem of knowledge as part of ongoing critical thinking about science and policy. In this view, science must be seen as a complex (eco)system for which the “rules of the game” change over time as society “evolves” (e.g., changes in political, social, and physical laws). Consequently, we must also be cognizant of emerging biases and inequities, and so develop a practice in ethics of sciences and as an “ethic of ethics” based on a community-based empowerment ethics practice (40). Potter (41) highlighted the importance of supporting such collective ethics of science as a frame to determine what is “feasible” (i.e., the right knowledge and technical standards). This practice of ethics should thus be understood as a form of pragmatic “action-ethics” grounded in a strong critical thinking process that emerges from people and the social. This action-ethics practice combines several approaches proposed by empirical bioethics scholars (26, 29): “inter-ethics” (42), “in-action ethics” (43), and “ethics ecosystem” (44).

## The Framework

### Assembling the Methods

**Figure 1:**
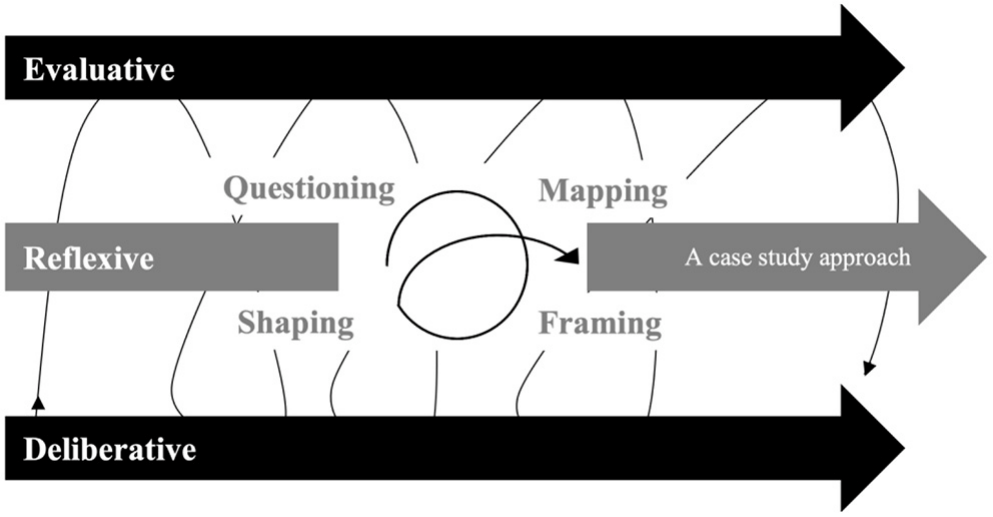
Framework in research and innovation to building reflexive governance for the responsible development of systems of information and communication technologies, hybridizing three types of reasoning are key to “reassembling” scientific paradigms in practice: 1. Evaluative as descriptive and traceable, 2. Deliberative as appreciative and prospective, and 3. Reflexive as knowledge balancing (1–2) to responsible policy. Reassembling is a “complexus” process (literally “being woven together”), here seen as an adaptive cycle and through the lens of the Huxtable and Ives framework in bioethics to support project management: 1. “Mapping” the ideas to generate a hybrid ethical theory, 2. “Framing” to be reliable to a case, and 3. “Shaping” a proper (micro) normative theory for change. We highlight the importance of ever questioning the whole process.

To build reflexive governance for SICTs development, we propose a community-based action-ethics methodology. The methodology bridges expert evaluation and collective deliberation in a way to empower individual stakeholders and engage group leaders (Figure 1). Project management in *Research and Development* must shift to a more integrative practice, coined here as adaptive *Reflective-Evaluative-Deliberative* cycle.

Evaluation (literally “the action of defining the value of”) is a key technique for bridging philosophy, science, and society through practical reasoning methods. Evaluation and deliberation must be conceived as independent methods given their distinct philosophical criteria and end (epistemology and teleology). To do so, reflexivity tools are crucial to avoid logical fallacies and to deepen and enrich the program value. However, these three methods benefit from being driven in parallel as interdependent methodologies (23, 42). Therefore, the expertise in One Health should be based on more advanced knowledge of social science and philosophy, which should lead to an applied “social ethics.” The bioethicist can help bridge political, social, and scientific insights within and outside organizations (42). These insights must be based on empirical observations and expert advice, namely a “co-construction” perspective hybridizing qualitative methods such as Delphi, concertation, participative study, and focus groups^3^.

Deliberation (literally “to discuss collectively in order to decide”) is a common reasoning process in day-to-day life. Deliberation aims at collectively examining, justifying, and questioning reasoning. However, political and professional deliberations should be about ethical justification, not scientific or political ones, or any private interest. Deliberation can be based on critical and evaluative reasoning, which would lead to a (*disciplined*) form of “collective ethics.” Collective ethics could become an end for deliberation. Deliberative reasoning has a foot in the political and scientific “arena,” both of which may or may not be conducted ethically. Collective ethics seeks to establish “ground rules” as starting principles that are analyzed from multiple angles (all stakeholders), including from the sciences and society as a “hybrid forum” (14)^4^. Deliberation benefits from qualitative methods (including those mentioned above), especially when rooted in political and humanistic approaches, such as hermeneutics (42). However, deliberation must not intend to “test” ideas or even describe a group's narrative, as would be the case of these methods in empirical sciences. The aim is to *reassemble*, seek consensus and acceptability on the proposal, and deepen the collective reasoning. Expert understanding and scientific knowledge must be mobilized in recognition of their descriptive and analytical value during such reflective processes.

Reflexivity, as the quality and method for critical thinking, connects the real world (empirical) to abstract reflection (intellectual), allowing feedback from both sides: evaluation and deliberation. But reflexivity is tied to the (epistemological) challenges of both philosophy and science, to which pragmatism provides some answers. Judging reflexive thoughts is a matter of dialogues, integrity, and trust, even of bidirectional relationships, of a continuous search for consensus, and of collective duty to empower people to question these “thoughts.” Expertise from philosophy or science must be careful not to become a (normative) dictature (40), even under urgent calls for sustainability, precautionary, solidarity, and responsibility in public health (11, 48–50). The competency of experts must be balanced with humility and compassion (51), or localism and experimentalism (52). The objective of reflexivity in pragmatism is to find *ever better* courses of action, the quality of which is established in light of the *future* (feedbacks). This “prospective science” is intellectualized as experiential learning (yet past), with the aim of archiving the common ground for cooperation, such as a vision, models, and theories (even formal agreements) of change (5, 39, 53).

### Apply a Balancing Approach

Following Huxtable and Ives' (26) framework in empirical bioethics, the process is divided into three phases to organize the reasoning:

1. “Mapping” the ideas to generate a hybrid ethical theory,
2. “Framing” to be reliable to a case,
3. “Shaping” is a proper (micro) normative theory for change.

In *A Companion to Bioethics* (54), John Arras understands this *framing* in bioethics as “casuistry,” an approach in ethics: “the [technics] of applying abstract principles, maxims or rules to the concrete case.” The “empirical case” (as used here) is a social collective on the edge of transformation, meaning to be reorganized or “reassembled” according to Latour's work (16) based on a unifying issue, here antimicrobial governance and digitalization. This Latourian perspective embeds the observer in the systems he is observing, which opens the possibility of a case study in empirical bioethics, focusing on the (intellectual) system of values characterizing and contextualizing these observers (based on a Ph.D. project in bioethics). To propose an ethical strategy for “managing” this system of values, which means deepening and seeking consensus among various interests, we adopt the logic of adaptive cycles in management, the one that gives rise to the perspective of adaptive governance (55).

Critical reflexivity is understood here as “balancing” reasoning. Reflexivity is a negotiating process between empirical data from interdisciplinary methods and rational insight from philosophical methods in order to find feasible solutions and long-term acceptable actions (6, 23). “Balancing” means having facts and values as two samples for which we do not know *a priori* their weight. We must experience this balancing (as an ongoing process) to ground the normative knowledge that should support decision-making. “Fact” means knowledge and empirical observations (*in situ*: resources, capacities, power, and will), while “value” is about philosophical questioning and self-critical reasoning. Values are given to qualifying, notably uncertainty, bias, even the usefulness and successfulness of decisions (56). Theories of value, as academic knowledge, archive notable critical reasoning paths. However, values, criticism, and reflexivity are also subjective.

Reflexive balancing, coined by Ives (23) with Heather Draper (25), is an ethical (meta)analysis combining methodology for interdisciplinary and critical reflexivity. The outcome can support self-governing processes that empower the community. At the collective level, *reflexive balancing* must begin at the start during initial planning, sampling, and questioning (Figure 1). This upstream ethical reflexivity and expertise have included the active role of FMVUM team members to conduct the interdisciplinary methodology. This means diversifying the disciplinary assets at the start; here meaning expertise in ethics, laws, technologies, and medicines, and more broadly community representatives. Note that facts and values are not dependent—as in “reflexive equilibrium” (23, 57)—but interdependent through the reasoning process. For example, sometimes facts justify actions that go far beyond what is acceptable and thus go against accepted human values; their application changes the “rules of the game.” Alternatively, values can justify change prior to evidence, as articulated in the principles of precaution, solidarity, or responsibility.

Social negotiation, a key concept of the deliberation reasoning (58), can support ethical analyses when applied to the evaluation of multi-actor systems of values as a “collective thinking process.” Ethical negotiation is enriched by co-construction approaches conducted at all steps of the program development and by acknowledging multiple scales of translation: here the expert, team, and community (14). At the expert level, the approach of Abma et al. (42) to bioethics was used to interact with actors (the Ph.D. student as a formal member of the FMVUM team) to deepen their understanding and positioning. At the team level, Samuel et al. (44) coined the model of “The Ethics Ecosystem” to empower stakeholders in the development of the governance system structure, functioning, and purpose, notably the influence of allocation of financial resources, conflicts of interest and shared responsibilities. At the (collective and biotic) community level, Ives' (23) methodology for empirical ethics provides guidance on how to negotiate stakeholder values and avoid fallacious reasoning through a reflexive, interdisciplinary, and pragmatic balancing method.

In summation, how to connect reflexivity and deliberation in One methodology with One (collective) goal? Where should ethics occur in decision-making processes to empower critical thinking from individuals to the whole organization? Is it by the mandating government (a department, e.g., MAPAQ), the responsible team (within the university, e.g., FMV), or individuals (representatives of private interests)? The three kinds of reasoning—reflexive, evaluative, and deliberative—integrate distinctive approaches from empirical bioethics into the project management process (Figure 1). All three steps refer to methods that have distinct philosophical standpoints (i.e., qualitative criteria of scientificness and ends). For instance, deliberation is about collective discussions and consensus-seeking, while evaluation refers to critical analysis (scientific, political, economic, etc.), but must also be ethical, meaning self-critical reflexivity. This linkage implies the opening of a dialogue “in the field” between experts, here in veterinary practitioners, epidemiology, data science, technology governance, etc., with different social perspectives (professional orders, industrial associations, interest groups, universities, government, etc.). In the spirit of the community-based action-ethics methodology, the intellectual assemblage must be conceived prior to the case study as an applied framework (Phase 1: Mapping). The goal is to anticipate core issues, first by thought experiments (59). Pragmatism, based on the relationship between theory and practice, focuses on courses of action and uses reflexivity to question and advance them (phase 2: framing). The outcome of pragmatism is empowerment often based on communication and/or education tools (Phase 3: Shaping).

## Applying the Framework

### Mapping

**Figure 2:**
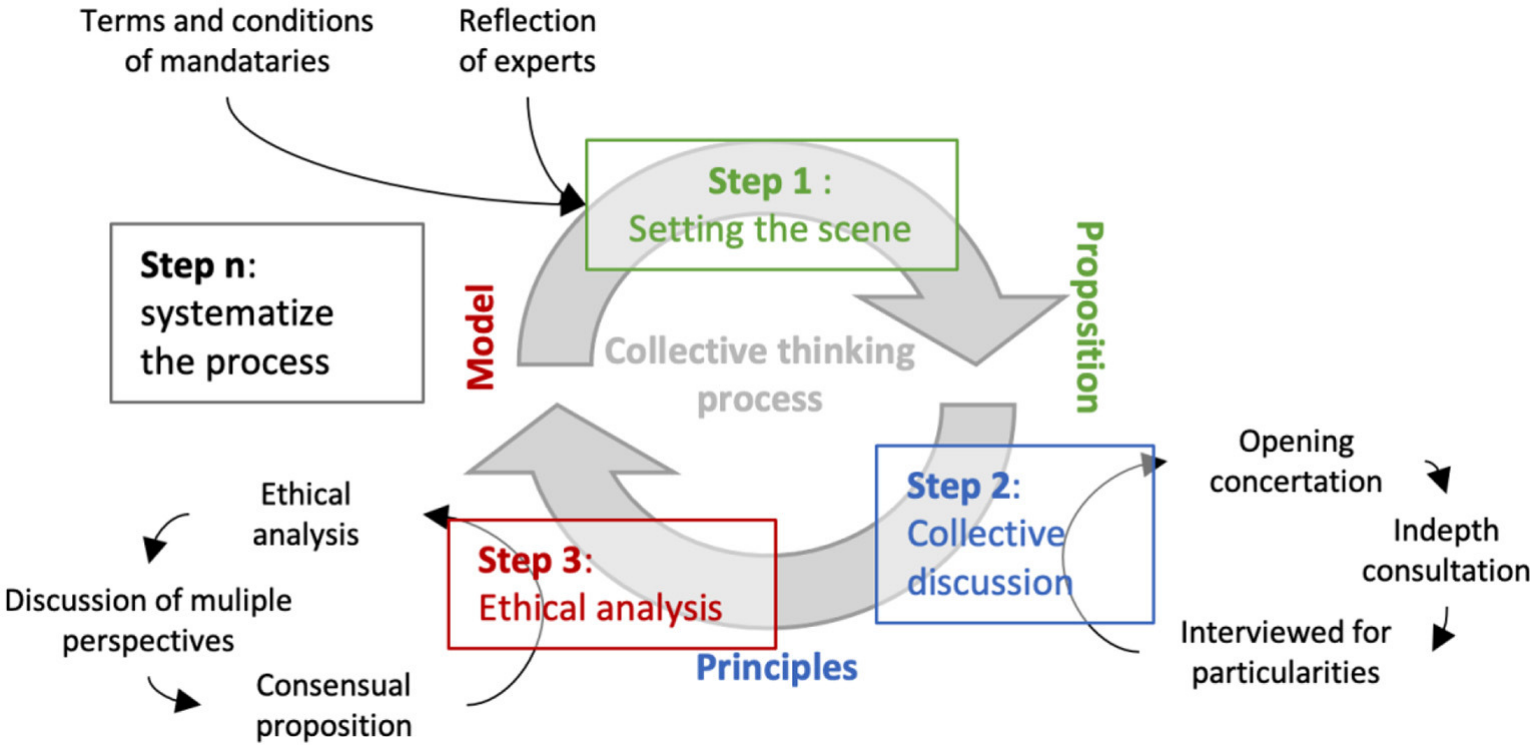
Assembling the methods for collective thinking to orient the project of a data and information system for antimicrobial surveillance in Quebec: A decision-making process that locates ethics by default at each stage requires a *reassembling* of the program development cycle of conceptualization up to implementation and evaluation. This decision-making process becomes community-based by the bridge it creates between deliberation (Step 2) and reflexivity upstream, i.e., before and during the conceptualization phase. This “preparatory phase” becomes a process of planning (Step 1) and evaluating (Step 3) decisions in order to co-build collective ethics (for/by the community) that empowers individuals to think critically and the community to critically evaluate based on dialogue, concertation, and a proper environment for knowledge translation.

Deliberation is part of decision-making processes and in the *right* “location” to position ethics in governance. Mapping means here, first, acknowledging an adequate theory in ethics (see above A Pragmatic Bioethics Approach) and, second, choosing the *best* deliberative strategies to put it in practice (see below). A hybrid methodology that connects reflection, deliberation, evaluation and decision in a systemic process is detailed in Figure 2. However, deliberation can become a way to scale up critical reasoning at the social level if mobilized for a collective ethic, rather than to plan the technicalities of the operationalization of a project. To be systemic, such a process must “exist” as a core functioning process of a community. Although to be effective, deliberation must be constructive and useful for stakeholders. Ethnographic methods can be used to plan and design the fieldwork to ensure the deepening of deliberative reasoning (60).

This reflexive “roadmap” with several checkpoints (Figure 2) guided the feasibility study (2018–2021) conducted in Quebec, Canada, to evaluate the possible implementation of a monitoring system for the use of antibiotics in veterinary medicine. The deliberation process initiated in 2019 with a “Proposition” (Step 1) toward in-depth consultations and interviews to coin collaborative principles (2019–2022, Step 2), as well as the work of expertise to “engineering” a model (2019–2022, Step 3) to ethically negotiate at multi-scale (micro vs. macro) perspectives on conflicting discourses (61). Such roadmaps are useful for strategic and ethical leadership. The iterative process is key to building leadership in a system involving shared responsibilities between coordination (the team), several stakeholders, and policymakers. Leadership is about positioning and evolving, which means giving a clear, yet visionary, position about: Who has a job to do? To what end? and For whose interests? (34, 62, 63).

#### Setting the Scene

Setting the scene (Figure 2, Step 1) is the first step for thinking about change (64). In the case study, this stage began in 2019 with concertation that brought together 60 stakeholders^5^. The “Scene” is about setting the “vision” (65) and guidelines for *good* “preparation” (66) and “regime” (32) for managing the change. This step requires *good* coordination of expertise, resources, policies, and infrastructure, and their proper methodological assemblage with theories, concepts, and principles: we need a “global roadmap” of the case “problematization” and then possible avenues for operationalization (67).

Maps or “normative knowledge” of any kind (e.g., laws, techniques, standards, and treatments) must be designed in close collaboration with the people to whom the norms apply. Norms must be co-constructed, emerge from large social collectives, and be deliberated through an adaptive cycle of iteration. Normative thinking will lead to strategy (e.g., action plans), but must first (Step 1) build on ethical analysis. Strategic and ethical thinking must eventually hybridize into one process of reasoning through iterative deliberation and learning from feedback (Step 2).

Potter and Lisa (68) critic “sustainability” for being too strategic and not ethical enough, and highlights a way out through deliberation. Deliberation constitutes a large and every expanding methodological field of study in sociology (69), participatory research (70), management (71), governance (72), etc., that we must build on. However, as the “last step” (Step 3), ethical analysis is crucial for questioning the “terms and conditions of mandatories” over the programs or its actions before “re-proposing” (Step 1) for deliberation (6, 66).

#### Collective Discussion

The collective discussion consists in bridging the general with the particular (Figure 2, Step 2). In particular, deliberation requires a systematic intake of credible information from *the field*, such as “consultations” and “interviews” could provide, which describes the community and its understanding of the change at stake. In that sense, deliberation needs to be *data-driven*, but not exclusively, because it must also be *knowledge-driven* by a process of transfer and “translation” (14). In general, the deliberation must be based on a vision of the change, like the one depicted in the “opening concertation” (Step 1) as well as transferable information from the academic literature.

The purpose of deliberation must be about governance: its architecture and future. For example, the case study began by deliberating on the form and content of the technology (2019–2020), then on the value of its SICT program and development (2021), and (to come) on norms for responsible conduct. Governance must be evaluated and deliberate at each “start” (72). This means building a dictionary from technological terminology to ethical meaning (Step 3). These last components of governance are immaterial but essential to envision a collective future (52). Meanings must be standardized (a dictionary), but also critical (reflexive governance) to learn and progress (36).

This mindset refers to system thinking (73). In practice, system thinking implies pursuing the deliberation process after the “end” of a specific mandate (iteration, Step n). The emerging vision and models provide insights for sharing the responsibilities, which means to learn and setting an *ever better future*—the scene (Step 1). For example, by laying the groundwork for a new proposal on the formal perspective of an Agreement in Principle for Responsible Animal Health Data Sharing (2021–2022). This Agreement must be initiated by design to acknowledge the “right” principle to apply for *good* collaborative governance (32) without delaying the speed of the change process (74).

#### Ethical Analysis

Ethical analysis, as a way to qualify the *good* and *right* “with discipline,” must be at each step of decision-making processes (Figure 2). However, the disciplinary ethical analysis must be at the heart of governance programs (Step 3). Although ethical analysis must give the basic tone to reflexive balancing, reflexivity benefits from an abductive dialectic: to be tested by pair review, as a “discussion of multiple perspectives,” and case study, as seeking for a “consensual proposition.” In other words, reflexivity gains in value by the constant search for its democratization: aiming at spreading its methods, like critical thinking practices, and its result, the evaluated climate, resources, and capacities, in the case (Step 2). Moving from deliberation to the scale of a social phenomenon requires a solid reflexive “terrain,” as depicted in Paquet's work on collaborative governance (75). To emerge from various stakeholders, reflexivity needs an appropriate “Habitat” to express itself, such as research hubs, living labs, innovation hubs, or business incubators, among which Observatory on the Societal Impacts of AI and Digital Technologies (OBVIA) supports the development in the Québec public-private-academic “landscape.” To be inclusive, this process involved first defining problems collectively and deepening positions qualitatively (Step 2) but, therefore, an ethical analysis to progress this collective position (Step 3) considering social ethics, which “rules” may also need to be (re)set for a new “scene” in law, health, and technology (Step 1).

Ethical reflexivity is proactive in the manner of an adaptive and learning management process (76). In practice, the bioethicist's reflexivity and the team's expertise must be synchronized to scale up the (micro) personal insights to the (meso) collective, then (macro) social level (23, 42, 44). For instance, the bioethicist in this regard joins the FMVUM expert team which gives, therefore, a solid interdisciplinary ground to connect analytically with the social discourses in Québec animal health sectors. The bioethicist must shape and question the tools guiding the pragmatic negotiation toward an ethical (“pro” to “post”) position, without deciding himself the sense of that “final” positioning, which must rely on an ever-evolving collective ethic (Figure 2, Step 2)^6^. However, the collective position must take strength in the leadership of official entities (sponsors, e.g., government), but not rely on the “belle-parole” of consensus normative principles (see the distinction between normative and appreciative knowledge in the last section). The leadership of the team in charge is based on two justifications (Step n). In the short term, this justification takes its strength from the political legitimacy, for instance, the Government of Québec's GPHP statement: through the periodic renewal of the “terms and conditions” by financial, regulatory, or declarative means. In the long term, this justification becomes powerful, however, this appreciative “parole” depicts an acceptable future having the capacity to impel a culture of empowerment (6).

#### Iterative Process

Ethical analyzes understood as ongoing critical thinking processes are a key functioning characteristic of reflexive governance. The ethical analysis aimed to criticize and give purpose to:

1. Strategic plans [feasibility and acceptability (78)] developed collectively to negotiate social discourses (58).
2. Decision-making processes conduct at multiple scales—the expert, the team leading the project, and representatives (e.g., sponsors, group leaders)—as they choose what best insights are meant for the community and about the future of society.

For example, One Health's scientific questioning is about how to develop a strategic (feasible vs. acceptable), judicious (risk vs. advantage), and responsible (short vs. long-term accountability) surveillance program for antimicrobial use. Conceptual roadmaps are useful to nuance and negotiate the positions of the various stakeholders (i.e., perspectives, roles, and missions) about the meaning of what is *good*, to move toward a consensual justification and thus binding decisions. The usefulness of such tools is not in their mapping of social system complexity (79, 80), but in mapping the system of values, interests, and perceptions (81, 82). A mapping of ethical frameworks and normative theories is crucial for the ongoing questioning about possible biases and finding ways to manage these appropriately when and where they arise.

When assembled with bioethical methodologies or approaches to guide the bioethicist in the use of theories in practices, deliberative *maps* (as the one depicted above) became pragmatic analytical “tools” and could support professionals or other actors in their day-to-day decision-making. These hybrid *tools* can be designed for individual or collective use. These tools focus on structuring critical reasoning to get through complicated choices; they aim to identify, nuance, and contextualize tensions that transcend the decision-maker. These tools can also be seen as evolving *roadmaps* of the One Health paradigm and an advance in applied ethics (such a tool is proposed in the last section). When applied to a case, these roadmaps emerge from a confluence of expert and community perspectives as both have relevant viewpoints. However, roadmaps should evolve based on experience, not mere intuition. Real-world feedback is a core asset for the ongoing process of *reassembling* what is collectively conceived as a *good* change supported by intelligible methods *from the field* perspective.

### Framing

Applied to the case of a feasibility study in veterinary public health, in Québec, Canada, we will see how methodological innovations are implemented by people, institutions, and theories in constant evolution: a complex that “weaves together” three dimensions of “global” existence (structural, cultural, and intellectual) that we have called “community” above (6, 15). This sociological perspective on the organization of science in society clarifies that there is no new generation of “social” but always new forms of reassembled structures, functioning, and/or purposes (16). This communitarian perspective means that much of the power is distributed in the social (of which people are the elementary unit and society is the overall organization) than what would appear to be the case under a centralized or even hierarchical understanding of authority (22). Answers to the question “how do we democratize deliberation” must find clues in “how the social “perceives” its own normative theory” in a case study: the “collective ethics.”

#### Learnings From Sustainability

**Figure 3:**
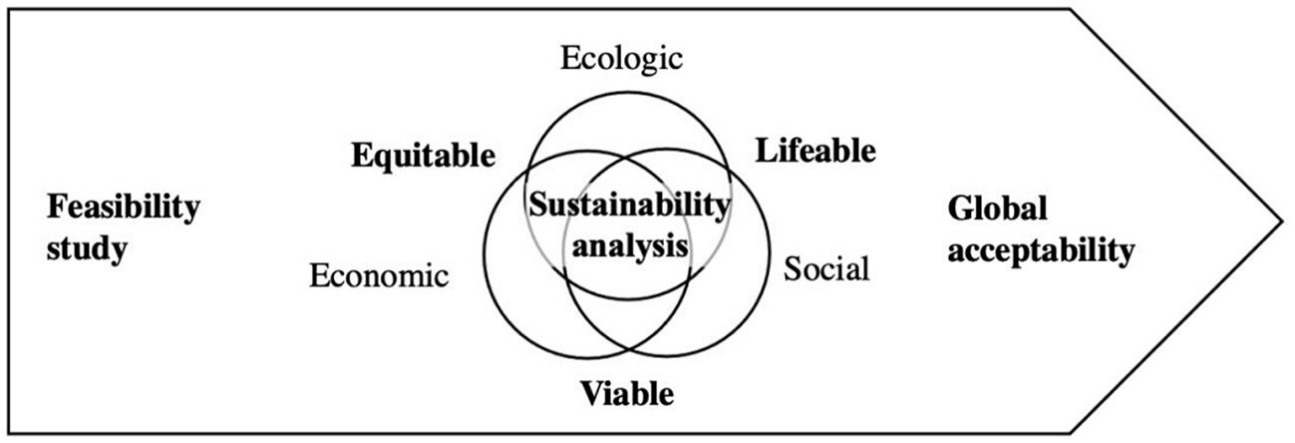
Responsible conduct for governing sustainable technosocial development [see Potter (6)].

A commonly used ethical tool for bridging reflexivity and deliberation in the field of animal health and environmental risk management is to refer to the pillars of sustainable development which, by their interface, bring out a set of values as emerging fields of study: *viability, livability*, and *equity*. These fields act as a driving force for interdisciplinarity, notably political economy and ecology (83). However, many scholars have criticized and advanced this approach, and from which One Health should learn (52, 62, 68, 84). For example, sustainable development aims to manage—i.e., (re)maps, (re)frame, and (re)shape—based on an ongoing process of balancing the value of short vs. long-term goals (26), such as mitigating the overall risk of resistance, with its short-term goals of health care services for local communities with antimicrobial governance norms (6). The sustainable analysis could become a tool to examine feasibility studies and manage its related ethical dilemma, for instance, short vs. long-term and private vs. common considerations. Even, it could be useful if it means degrowth as not-developing pharmaceutical or reduce the use—i.e., a reframing for global acceptability as a Potterian's socio-ecological concept (Figure 3).

The real world is also much more complex than these three pillars and values of sustainability, because the observer evolves, learns, and “thinks [s] in systems” (73). Norton, who works on the “philosophy of sustainability,” explained *good* development might be rooted instead in a “localism, experimentalism, and multiscale analysis” approach (52). Norton's understanding of sustainability allows for organizational resilience built on adaptive agility of collective policies: the adaptative management (85), allowing for several scales of observation, here the bioethicist, the FMVUM expert team, and the community as a whole (86). This learning ability is based on a functioning cycle: reflexivity, deliberation, decision, and evaluation. By reconnecting the cognitive and social spheres by introducing a multidisciplinary team at their interface called “Frontrunner,” Loorbach lays the foundation for an alternative model of governance that decentralizes this four-step thinking process within the network. Frontrunner's goal is to guide the transition management, notably toward sustainability and stakeholders' empowerment, for instance, about their digital environment, responsible conduct, and norms of practice on antibiotic use. Managing here means “reassembling” the decision-making process to clarify who decides, even more, who is accountable and expected to justify; and who evaluates, that is, who provides feedback, including useful learnings to improve programs and actions (87). “Reassembling” implies a dynamic planning process well before a crisis occurs, in order to quickly involve actors with short notice to “reorganize” in (infra)structure and culture (16). To implement these new sociological models with systematized methods, we also need to use collaborative ethics (36, 88).

#### Learnings From Governance Systems and Community

Community-based approaches refer to collaborative and learning mechanisms: Let us propose the idea of an “adaptive co-management of transitions.” Collaborative thinking is facilitated by systems of information and communication technologies (SICT) that scale data sharing to knowledge transfer, translation, and applications. The philosophy advanced in the case study was based on learning: antibiotic use surveillance supporting both national antimicrobial governance policies through public reporting and refining individual conduct through voluntary benchmarking.

Although there have been technical advances with SICT, an ongoing challenge in ethics remains to bridge the gap between experts and the community. A possible solution lies in critical deliberation and evaluation (89). Along this line of thought, Rüegg et al. (63) present an approach to collectively plan and evaluate sustainable health interventions by thinking beyond the frame to working on a strategy to support convergence and make change happen. In alignment with this perspective, Bordier et al. (90) assess the methodological and epistemological challenges behind the evaluation of knowledge emerging from “multisectoral collaboration” through “interdisciplinary insights” (91, 92). Multisectoral collaboration needs to be evaluated, notably with a focus on the performance of the surveillance process (4), on its systemic process of sharing information (91), and on the consequences of the resulting policy (36). However, one of the most challenging aspects to evaluate is certainly the value, the credibility, and the validity of One Health knowledge and policies across different domains at the time of its justification to accelerate its application (18, 23). Some answers may be found in pragmatism and how the community can learn.

To ease the functioning of SICT, one of the upstream goals must be to develop a common consensual language that puts everyone “around the table” on the same “terms” (terminological and ontological). “Language” here means helping each person or group to move beyond their position and broaden their perspective to a collective reference point (epistemologically and teleologically). “Common” means a collective assemblage, joined under one “roof,” in constant “recruitment” of social actors, diverse (axiologically) in problems, abilities, and ideas, but all interested in managing the same “problem” (14). The case study bridges the emerging collective ethics of stakeholders and the social ethics emerging of collaborative governance theories (32, 72, 75) by deliberating on an Agreement for Responsible Animal Health Data Sharing. Governance is about administrative and political structures but also goes beyond this, as a regime and vision whose rudiments must be materialized in intelligible documents accessible to all, such as an agreement, charter, declaration, code, etc.

The case study highlighted the challenge of transforming a collaborative regime (as a philosophical theory) into a governance body (administrative practices) whose functioning is acceptable for stakeholders—i.e., justified by collaborative ethics—and still meets the expectations of sponsors, mandatories, and society (34). The solution found was in iterative processes: collective ethics and governance regime must emerge from an adaptive cycle of ethical decision-making processes and from empowering leadership materializing the process in action (Figure 2). This new cycle (proposed for 2022) will involve the signature of that Agreement, which will “shape” the development of the data platform. To ensure the trust of stakeholders from the start to the end of the biosurveillance programs, which will evolve to expand data input across sectors and the overall outcomes of aiming to implement the One Health perspective, the Agreed Principles must progress as well (post-2023).

As Abma et al. (42) outlined, bioethicists are key assets to co-evolving practices, as “inter-ethics” (for proactive and interdependent) bridging the gap between the leadership team and the community. *Inter-ethics* opens deliberation on the (intern) program and (extern) partnership policies and values. Bioethicists are key to the functioning of large organizations such as companies, research groups, and public services. The functioning transcends (as singular) the ability to foresee a decision-maker, as the Chief executive officer (CEO), even the Chief information officer (CIO). Hermeneutic approaches, such as the maieutic process of Socratic dialogue (93), or others from applied ethics, such as casuistry (case study), should be implemented as a day-to-day approach for improving the critical thinking practice (as singular: CEO and CIO), even allowing for collective and deepen deliberative reasoning (58). The bioethicist leading these ethical approaches should not *shape* the problem in practice (94), but support the process of intellectual *mapping* and interdisciplinary *framing* (26) to help stakeholders design and manage their ethical *shaping* process (40).

The role of the bioethicist is to provide an adequate habitat for collectively “thinking global,” not to prescribe global thoughts (23, 42, 44). As illustrated by John Godfrey Saxe's (1816–1887) “The Blind Men and the Elephant:”

*And so these men of Indostan*

*Disputed loud and long,*

*Each in his own opinion*

*Exceeding stiff and strong,*

*Though each was partly in the right*

*And all were in the wrong!*

The main criticism of global thinking, as here depicted in terms of communitarian approaches with the “elephant,” is about the sense of urgency: Is their time for this discussion between “Indostan”? Antimicrobial use calls for rapid and radical change, yet incompatible with cultural changes requiring long-term collaborative, democratic, and reflexive processes. In the animal health sector, urgent and radical change means the commitment of stakeholders who are complexly organized as shown by Majowicz et al. (79, 80). Moreover, long-term collaborative processes mean reconciling a diverse system of deeply distinct ethical values (45), notably the views of the agri-food industry vs. vegan activists, or even traditional indigenous knowledge (34, 95, 96). The case study shows that co-building a collective ethic tends to accelerate the commitment of stakeholders by linking their actions to an awareness of the consequences, which leads them toward a culture of change. Collaborative governance, here defined as a state of mind (72, 75), even a community-based approach (97) or a communitarian paradigm (22) rather than an (administrative) governing body, accelerates this cultural bridge to the future (5). However, to be collaborative, governance must also acknowledge specific and generic concerns: sometimes even questioning collective paths in the face of local issues (bottom-up) or front of societal values (top-down). This abductive process (“local-to/from-global”) necessitates the integration of top-down (e.g., government to citizens) and bottom-up (e.g., citizen to government) modes of governance.

#### Learnings From Pragmatism

The case study was built on a pragmatic approach to ethics. Whereas, the *good* relies on imperatives (yes/no answers) in a deontological or legal perspective, the pragmatic ethics approach shifts the emphasis from the “imperative” to a deeper collective deliberation process that democratizes thinking about such ethical criteria as *good, right*, and *better*. Pragmatic ethics recommend, first of all, putting aside the prioritization of which is “right” or “wrong” between scientific, traditional, and alternative knowledge and beliefs (87). The priority is to act on tipping points, such as the need for surveillance of antibiotic use and thus for the governance of its SICT to refine practice, by seeking consensus between the parties involved on how to do it ethically (18). In practice, Callon (98) named these as points/nodes of common problematization and explained how convergent perspectives and interests stimulate the recruitment of ever-increasing numbers of actors around the common problem to solve. This is about assigning duties for what and to whom, i.e., the “pragmatic sharing of responsibilities.” Moreover, responsibility is linked to resources, so deliberations must focus on the actor's duties, assumptions, and capabilities: does each stakeholder have the necessary resources, opportunities, networks, technical abilities, theories, or other necessary “tools” to achieve their goal (their responsible mission)? In the case study, all the actors involved—industry, activists, and researchers—agreed on the importance of solving the antimicrobial resistance problem, even if it was for different reasons, and to archive this collective position in an Agreement, whose principles would detail these contextualized duties. Under this pragmatic view, the core problem was no longer the “Why” to act, but the “How” to interest all stakeholders to act collectively and in concerted fashion, i.e., the “common problematization.” This led us to apply ethics and its rationale to share responsibilities appropriately (e.g., duty, ability, and capacity to act) among the key stakeholders.

One of the core challenges of One Health is to operationalize pragmatic processes (the “how-s”) and build consensus for action. Indeed, some deontological positions are inevitable (the “why-s”) and create conflictual ethical points of view (18): Who should be responsible? Which core values to prioritize? Is it for the benefit of humans (anthropocentrism), all living beings (biocentrism), or communities (ecocentrism) that we should act? Mermet's work on social negotiation (58, 69) can help bridge the gap between *Social* and one's *thinking*. Designed for strategic analysis, social negotiation can provide pertinent tools to bring into practice Latour's framework [see Bilodeau and Potvin (99) in public health].

However, strategic analysis is a “descriptive to normative” knowledge translation process. This translation must be combined with an ethical analysis to prevent fallacies. For example, medical diagnosis (prescriptive) must be based on history and Biology (descriptive), but overall, the transition from one to the prescription of a particular treatment (an antibiotic) must be based on the clinical judgment of the physicist. The action-ethics framework presented here proposes such a “descriptive/appreciative-to-normative” knowledge translation process applied to political processes. Defining the *right* course of action, meaning the “justify normative knowledge” that will lead to responsible actions, requires the involvement of many people from various disciplines. Appreciative knowledge is the key to expanding the perspective, for example, the Government of Canada's Categorization of Antimicrobial Drugs Based on Importance in Human Medicine, which is normative, seeks to prevent harm by adding such antimicrobial governance insights to medical practice. The “inter” of the “interdisciplinary” is about quality: Who or what is bridging? Too often, the “appreciative” fields of knowledge, carried by the humanities or the human sciences, are underrepresented. Co-building collective ethics as a structuring process of a One Health transdisciplinary program will help to highlight, “in action,” those missing pieces for normative practices.

An important criticism of pragmatism has to do with relativism. Who actually decides what is *right* after all? Notably about data access or even antimicrobial governance. Is it the Law, the people, the market, the activists…? In terms of relativism, these dimensions—legal, civil, financial, associative—pose “truths” of equal importance. Pragmatism in ethics does not, in any way, reject the importance of deep debates, nor the negotiation of these dimensions, or the radical questioning of the way things are done (18). Pragmatism cannot be achieved without these in-depth reflections to define broadly which *better future* we want to achieve collectively (5). Therefore, pragmatism is more about deliberation than decision, even more about education than action. However, the main characteristic of pragmatism is, indeed, action-oriented: deliberation and education process must lead to tangible, practicable, and (if well-done) prospective knowledge—such as collective vision and goal. Such an objective for discussion leads to determining agreeable points to act at a specific time and place and acknowledging a need for an ongoing process of evaluating, criticizing, and adapting those pathways of action. These enable progress for/by the community while recognizing potential harms to individuals and the environment (suffering, vulnerability, and existence). Giving credit to the community, pragmatism justifies having representatives (as an expert or social voice) capable of deepening and raising positions anchored in complicated scientific phenomena and complex system values.

To know who decides, the question should be: “Who is the most credible to carry out the collective work of deliberation?” and more importantly, “Who is responsible for it?” The case study showed that multidisciplinary teams mandated by public authorities can become key actors to structure transdisciplinary projects—a *Transprogram*—as a “flying team” in the collective creating a dynamic bridge between the expert and the whole (46). The team becomes a binding, critical, and justifying force: public values (democratic government), academic knowledge (“balanced” expertise), and a “targeted” community. A “transprogram”—a neologism that implies a “transdisciplinary” in action (56, 89)—can be conceived as a continuous process of knowledge building and collaborative governance (16). This forces us to complexify our understanding of the “theory to practice” challenge. It is not simply a question of bringing knowledge to action through “communities of practice” or other forms of collective (100, 101). It necessitates theoreticians (e.g., philosophers and mathematicians) develop the “practitioner” reflexivity personified by *in situ* questioning of what we must do as a person and how to empower such critical thinking.

We need to organize what we—as a collective—are saying. Transdisciplinary—as the increasing relationship between sciences, technology (e.g., the industrial products), and society—introduces a confusing mess of terminologies, methodologies, and philosophies that must proceed throughout the program (as political, scientific, and societal) development. At a minimum, proceeding with this “mess” in practice requires ethics: critical thinking, codes of conduct, and responsible organizations for an ever-learning process in ethics. Extended to society, a postnormal philosophy of sciences (56) proposes new models that recognize the value of falsification (102), but extend the theory about the *Structure of Scientific Revolutions* (103) to include new tools from sociology, anthropology, and technology emerging from the digital age (74, 104). As presented in Figure 2, program development must return to the scale of people (theoretician, practitioner, “fieldworkers” and representatives) but be institutionalized as a democratized deliberation process. The challenges of such collaborative governance (72) and transdisciplinary research (105) are to be contextualized in a constantly changing world without losing the local perspective as developed in Morin's complexity paradigm (see the synthetic tool in the next section).

A pragmatic bioethics approach will be crucial to achieving this goal. To seek precision, the sciences tend to fall into the specialization process (disciplines and techniques) and lose the “big picture” as Saxe (106) has noted. On the other hand, philosophical reasoning, methods, and theories in ethics may lack an operational strategy for seeking and driving empirical and practical change toward empowerment and political sharing of responsibilities. Both aspects need to be integrated into an interdisciplinary process to proceed to a sustainable course of action, and this is where bioethics can step in (107), to act as a translation mechanism, and so become the missing link to materialize interdependency (37) without resorting to disciplinary reductionism (108).

### Shaping

**Figure 4:**
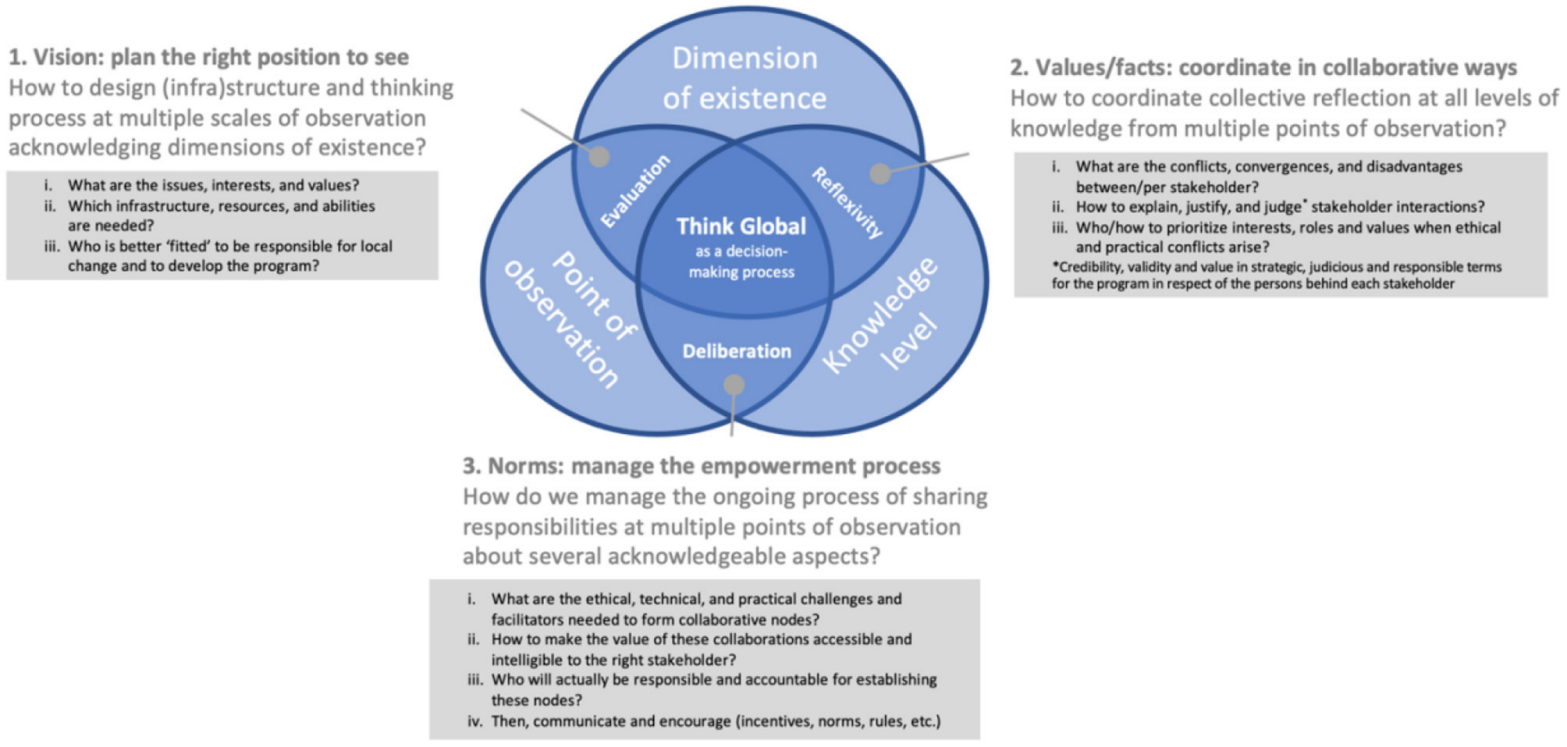
Framework to broaden critical thinking used in the case study to building reflexive governance: A bioethical tool, “mapping” One Health methodologies and teleologies, to help in “framing” and “shaping” the work to deepen the meaning of persons, groups, and the community reasoning to broaden the collective perspective and co-build a global vision, but still locally collaborative, of One Health governance programs. Designed in empirical bioethics by case study approach, in the Quebec animal health community, the one emerging from the commonly shared One Health problematization of antibiotics (production, use, and resistance) linking Government Ministries with shared jurisdiction over animals, human, and environmental health, this tool seeks to broaden the critical thinking of the expert training in bioethics, the coordinating team members with leadership, and stakeholders. Constructed by abduction based on the revised theories and the case study depicted as a community-organization that evolved its practice. This tool must guide the reflexivity of each person at each of these collective discussion steps.

How can we seek Global acceptability? How can we mobilize science paradigms to set a “feasible” normative theory leading to a co-built code of ethics for empowering the community? (6) Shaping ethical tools, such as codes, methodologies, and education resources, are core assets for sustainability because they pave the way to basing its operation (the result of integrating the three pillars) on values (e.g., its equity, liveability, and viability): each action must be rooted in in-depth justifications (the values) bridging sciences and ethics (5). One Health benefits from such tools, here called “bioethical” referring to this Potterian “bridge,” in the format of reflexive, deliberative, and evaluative practices. The bioethical tool below contributes to deepening methodological reasoning to guide toward more practical pillars, but still rooted these in the core values of sustainability (Figure 4).

Broadening the vision in Saxe's poem means being more integrative, even appreciative. These *levels of knowledge* are about building consensus and deepening the reasoning of each stakeholder from their own *point of observation* to define the problem and interest in its resolution. A vision of change must transcend all *dimensions of existence* to question fallacious reasoning all along with the life of the surveillance program coming from integrating *levels* and *points* (26, 42, 44, 105).

Figure 4 proposes three practical questions at each interdependent interface—(1) evaluation, (2) reflexivity, (3) deliberation—to share responsibility and “take a position.” First, “plan the right positions to see,” as an *evaluator*, in terms of infrastructure and paradigm, and then “coordinate the scene and assemble collaborations from these positions” and diverse knowledge (several perspectives per level). For example, to see and coordinate, search for, or create a governance body with authority over financial planning, institutional sustainability, and stakeholder accountability (36). Collaborative governance increases credibility and trust (32), especially when the partnership that embodies this governance diversifies the collaborators. Then clarifying the *rules of the game* facilitates comanaging the empowerment process in terms of “adaptive governance systems” through deliberative processes. Taking a position means balancing knowledge from experts and value from sponsors [formal and informal terms and conditions (23)]. Note that (e)valuation is a continuous ethical-scientific process that must be pursued throughout and collectively on as many sub-dimensions as possible, and involves balancing the three identified here for a valid (pro/post)position (green) and the trans-level of knowledge (red) in time and in forms that support decision-making.

Evaluation (1) should be a continuous process, as the concept of surveillance and program evaluation understand it, but also includes the observers as a thinking unit to study the impact of the technosocial initiative and its policies. Observations should be planned upstream, based on both understanding and vision, to locate the *evaluating*-observer in an ethical and strategic place to see. Vision means a roadmap of what is foreseen as *good*, envisioning a *better future*. Maps are factual models built on-premises (values). Deliberation (3) as a social negotiation process is crucial to translate knowledge, vision, and values into policies. Deliberation implies negotiating the interests of the parts, judging actions, norms, and singular values in front of the common interests. Reflexivity (2) is the solution to an ethical, pragmatic negotiation.

The bioethical tool (Figure 4) integrates the three pillars to “Thinking Global” (existence, knowledge, and observation: Table 1) describing the intellectual world in which evaluative and deliberative decisions must be made:

1. Dimensions of existence (the empirical): the experience of life as a person and as a collective (family, society, and humanity) whose experience and context become accessible from the attentive “eye” and reflective strategies conducted in a community as a space/time, transgenerational and multispecies concept (22)—“What exists?”
2. Levels of knowledge (the cultural): sophisticated tools ranging from technologies to theories helping to deepen reasoning, to justify decision-making, or directly to change the conditions of existence—“What should it be?”
3. Points of observation (the intellectual): the intellectual lenses leading to subjective action of the expert and from the community (as thinking units, not things) to assess the effects of decisions on several patterns and scales—“What do we want to do?” “What could it be?” and “How will it be done?”

**Table 1:** Reflexive aspects to think “Global” about, before and while planning and deliberating on the surveillance program^a^.

	Definitions^*^	Reflexive questionings^**^
Dimensions of existence	Empirical experience: what have we experienced as human (the subjective point of observation) and expert (the person deepening the knowledge)? **Three existing worlds globally posing complexity** 1. Biological: the physical dimension of life 2. Sociological: the institutional dimension, including laws and culture 2. Anthropological: the intellectual dimension and values (e.g., health, well-being, biodiversity)	**Localism as “To think” per community** **Sustainability**: How to strategically distribute the “observer” reflexivity and evaluation to integrate multiple dimensions of existence, to assess several organizational scales, and to judge biases and prejudices over time?
Levels of knowledge	Cultural learning: what have we learned through history (the human existence) and as communities (the overall existence above)? **Three existing thoughts posing human complexity** 1. Descriptive: understanding of the cognitive and surrounding world (to acknowledge the above dimensions of existence) 2. Normative: systematized course of action, e.g., laws, techniques 3. Appreciative: thought qualifying the past, present, and future	**Experimentalism as critical questioning** **Acceptability**: How to judiciously choose the “right” knowledge to the proper end, to integrate learning, to engage the community, and to critic decisions constructively?
Point of observation	Intellectual critics: How to criticize each other's positioning and abstract collective actions? **Three existing states of organizational complexity** 1. Networks: interactions between actors and their environment (see the actor-network theory) 2. System: a dynamic assemblage of several networks evolving according to their own principles (see the concepts of social collective or ecological community) 3. Organization: an open system with various alternative states of succession remaining stable through retroactive processes of self-determination (see the concepts of biological organism and ecosystem).	**The multi-scale analysis deliberating process** **Responsibility**: How to ethically manage program development to improve transparency in governance, arbitration of resource allocation, transition of cultural change, progression of decision-making, advancement of collaborations, and communication in the manner of a community-based, adaptive, precautionary governance process?

a *Referring to the Morin's paradigm of “human complexity” and “Penser Global”, applied to One Health, from a synthesis of complementary theories, notably Max-Neef, Latour, and Ingold work on the translation, organization, and evolution of scientific and traditional knowledge. This synthesis is rooted in Potter's bioethical normative theory and approach for pragmatically bridging Sciences and Society to reach the goal of improving toward a better future. As any “shaped” map (technological, geological, or ecological), the landscape is in motion which requires having the case and its context under study*.

* *Scientific paradigms must be used to ensure that collective ethics is shaped within the frame of sustainability (the “what is feasible by nature”) to broaden our understanding of the case study (29). Built on values, the purpose of this synthesis is to broaden the vision to set ever better ethics to guide conduct, policies, and governance processes toward responsibility, i.e., the practice of empowerment ethics*.

** *The synthesis was translated into questions to ease their use in situ. The purpose is to broaden the collective vision of a common change for better policies and governance processes by building a program based on core values (sustainable, acceptable, and responsible) that emerge from deeper reflections on what is “feasible.” Values must apply to the ethics of science (e.g., methodology, scientificness, and accountability) to improve evaluation practices throughout deliberation and reflexivity in program implementation. This helps to justify advanced surveillance goals and processes based on a broad vision that is anchored in the paradigm of complexity (15, 109), using the precautionary principle to justify action before a causal mechanism is fully understood, such as in the case of climate change, biodiversity loss, and antimicrobial resistance*.

#### The Dimensions of Existence

The bioethical tool (Figure 4) extends the scientific perspectives of “experiencing” the *existence*—the observable (objective) and the being (subjective). Physics, for instance, is not only a scientific discipline but also a *dimension of existence* (15). As a knowledge, *Physics* describes the complexity of the physical world (from the Greek “physis” means nature); thus, giving physicomathematical architecture to science paradigms toward natural laws, notably in chemistry, biology, pharmacy, and also engineering, medicine, management, and any kind of evaluation techniques on antibiotics or environment (110). As a dimension, *Physical* is about space and time from which empirical phenomena emerge; thus, providing (predictive or reflexive) insights—the pharmacokinetics of antibiotics, the probabilities of resistance genes, the microbial ecology studying natural evolution, and ecotoxicological geography of heavy metals (111).

Physics, commonly referred to as objective, natural or factual understandings, is one of the fields of *descriptive knowledge*, but the “empirical” is also about the collective existence in that *physical* world, involving norms and standards. Some *normative knowledge* is based on the empirical description: when observations become physical laws through experiments or even when these laws are translated into strategies, techniques, and technologies through the lens of understandings and values, for example, a standard based on the “ecosystem services” or “footprint” communication tool (112). Although mechanical laws and probabilistic models translate the physics of the world into understandable terms and tools, it does not mean that the whole physical world is, at some point, entirely understandable or even partially controllable (the positivist fallacy), especially when it is necessary to cross dimensions (the Morin *bio-socio-anthropological model*), such as the psychology of antibiotic users and the ecology of antimicrobial components. Recognizing this fallacy, *descriptive knowledge* must not prescribe actions on its own—e.g., the statement: “This antibiotic will cure that disease”—without being understood through the lens of scientificness (e.g., validity) and as human power and will, interwoven with belief and values, and embedded in conflicting interests and missions (45, 56, 87).

Alongside *Physics*, several other dimensions make it possible to analyze humans within their own *existence*: the *Social* and *Anthropological* dimensions of life. As for *Physics* vs. *Physical*, all those 3 dimensions are related to knowledge, among others, in psychology, ethnology, and axiology, which gives us access to its perspective. From those perspectives, we—as humans and humanity—experiment by observing and being: the dualistic (objective vs. subjective) experience of life (113). The need for both *Object* and *Subject* perspectives explains the usefulness of integrating natural, social, and human sciences. *Subjectivity*, to be understood here as *reflexivity*, brings the missing piece to positivism: the so-called postpositivism. Values, the missing piece, respond to uncertainty (56). For instance, political decisions on antimicrobial use and the progress of science in pharmacology and ecology must be proactive and responsible (the precautionary principle), despite there being no evidence (at least yet) on all the mechanisms of antimicrobial resistance, nor a full understanding of microbial evolution (1).

The challenge is to “Think global”: How to integrate all these dimensions, acknowledging the pluralism of perspectives and values? Moreover, how to progress decision-making with critical reflexivity, but without rhetorical fallacy? The answer points toward deliberation and evaluation to deepen everyone's positioning. This process must emerge from science and society (e.g., the Intergovernmental Panel on Climate Change, IPCC, or any public hearings, association, or platforms) as a community-based action-ethics methodology, although they will for this bridging process is not a given at the start.

#### Levels of Knowledge

Decisions are fundamentally subjective, because humans—expert and non-expert—are beings, not things, and think. Thus, some knowledge is more likely to change, while others are more stable over time (23). For example, the former refers to medical diagnostics (*appreciative*) and State laws (*normative*), while the latter refers to scientific observations (*descriptive*) such as those of physics (105). The speed of light and the gravitational constant are given (*fact*), while policies and diagnostics can change, and even less stable are the beliefs (*opinion*). These *levels of knowledge*, its strength, and even possible progression are not really *messy* but require an ongoing process of communication, management, questioning, and transparency to avoid fallacies (114). However, *normative knowledge* is a broad area. The decision leads to such knowledge: norms are about the *Act, Vote*, or any techniques archiving someone decision, which systematized action. For example, legal laws are *normative* as well as government, industry, and academic programs operating in the technology and, more broadly, into the *social*. Decisions—and the following actions—must always be studied, evaluated, and reframed collectively to progress these norms^7^. *Progress* is driven by examining the criteria and quality of its justifications (validity, credibility, integrity, etc.): the value of ideas and advances. These values become shared *appreciative knowledge* under community-based ethical analysis, which integrates academic, political, and civil perspectives as *Global* evaluative insights (16). The justified decision is about “responsible conduct” and “social responsibility” at the actor and network level, and should not be based on a decontextualized singular interest or ideology (5, 115). Democratizing governance processes through education and promotion refers to pragmatism or “collaborative governance,” and operates through communication, open dialogues, and constructive criticism on the justificatory and uncertainty value of programs (72), but implementing large-scale evaluative, deliberative, and reflexive practices remains, indeed, a challenge. Avenues for action have been highlighted here, including the organizational dynamics emerging from an Agreement hosted by a collaborative governance body and evaluated by a Living lab.

*Good* decisions and norms, which means being *shaped* by ethics, require more than being *fact-driven* or *value-driven*—they need both. This requires deepening the thinking process to “transcend” all *levels of knowledge* (105), that is, to bridge the *descriptive* and *appreciative knowledge*, as the Québec Agri-Food AI Ateliers has been a successful example (46). “Transdisciplinarity” implies going beyond statistical, mathematical, or predictive data-driven reasoning to interpret data and models ethically, as appears to be *a priori* monitoring of antibiotic use and *a posteriori* the translation of surveillance insights into antimicrobial governance policies. Classical scientific methods (positivism) are valid when framed by models or conducted within controlled environments, but fail in the real world, notably the ecological (*in situ*) surveillance of antimicrobial resistance (111). This issue opens room for reflexivity and deliberation in research (as *action-research*), but also more broadly in society (as *action-ethics*), requiring educational tools to operate: the example of the antimicrobial footprint (112, 116), which integrates learnings and competencies from history and art (see Saxe's poem, above). Hard decisions about human life, environmental crises, and next-generation implications need to be “based on ethical values, which are in the long run inseparable from scientific facts” (paraphrasing Potter's maxim). This intellectual agility requires transparency to challenge justifications prior to undesirable events. Seeking transparency must be a constant and proactive quest, becoming even the core (functioning) aspect of collective ethics leading to the emergence of empowerment practice. Although conceiving *how* to manage and acknowledging *what* such transparency might be complicated to assess, deliberation points to possible paths for action.

#### Points of Observation

One of the main aspects to be considered goes beyond knowledge and existence and enters the area of actions. This perspective, or *point* locating the *observer* in action in the world, refers to a “bridge to the future” (5) and is about “human responsibility” (117). The positioning, as the inspector, researcher, decision-maker, or even public health policy perspective, is in constant dialectic with (influencing it and biased by) its contextualizing system (73). Indeed, “Ethical values [the positioning] cannot be separated from biological facts” (6), meaning the surrounding ecology and economy of antimicrobial resistance to the inner psychology of behaviors and will of antibiotic users and decision-makers. Ethical values are an articulation of the (free) will to change. A will for change must emerge from the case (*in situ*) through convergence with applied sciences and practices, as initiated in One Health and sustainable development. Theories remain crucial to understanding what is observed, e.g., through the anthropological (belief, family, history), the sociological (institutional power and knowledge dynamics), and the biological (e.g., organisms and organizations).

How can we manage to *Think globally* while *acting locally*, as individuals within the (social) collective, (biotic) community, even (planetary) ecosystems? How do we evaluate locally (for us) while deciding globally (for all)? How do we do planning (long-term) while implementing (short-term)? How do we regulate (decide) while questioning the norms, guidelines, and understanding that have been established? (6) These questions find some solutions under the theoretical frameworks of “thinking in systems” (73) and mathematical scales (118) as “coadaptive management” and “adaptive governance” processes (77) and under more applied frameworks such as in “transition management” (74) about governance bodies and socio-ecological systems.

Deliberation is linked to the growing interest to find ways to integrate experts, traditional, citizens or, even alternative knowledge (71, 113). However, this should not reduce the value of scientific knowledge, but rather enrich it; these different types of knowledge (expert and non-expert) have different functions in the construction of human narratives. While expert knowledge seeks disciplines (laws, principles, mechanisms, and measures), other forms of knowledge express values, cultures, and beliefs. The latter communicates the realities of humans, beings, and things in various ways. Acknowledging the pluralism of values, as the appreciative knowledge of a collective, is a driving force (the free-will-power) for empowerment. These values can justify action before crises, i.e., to set in action the whole “scene” (Figure 2) to build the resilience of the system. In short, the will of a government or single decision-maker is insufficient to encapsulate the will of all (14): we need ethics (codes, methods, and prospectives) to empower each one to collective changes with a roadmap and a compass in order to navigate between different wills and aim at the common project (119). However, as an opening, this code must progress and go through an iterative phase of questioning (Figure 1).

## Conclusion

This paper seeks to lay the foundations for a methodological framework in empirical bioethics. Instead of focusing on ethical theories in philosophy or sciences, we reviewed the methodological literature in empirical bioethics, One Health and Sustainable Development study to lay foundations in pragmatism (J Dewey)—(*descriptive*) pathways to operate instead of (*appreciative*) guidelines to dictate (*normative knowledge*). The ultimate goal was to support the actual will in those fields of study to build reflexive governance, notably in One Health, to address the issues concerning the pharmaceutical agents necessary for medical practice (the antibiotic cure), but modifying the environmental conditions (the problem of antibiotic resistance).

To bridge the gap between person-to-person dialogues and social negotiation processes, the operational pathway goes through comanagement techniques and must target cooperation nodes. Notably, the manager must bridge the gap between the construction of the *Social* (its ethical narrative) and *collective* practices, which leads to empowerment ethics. This operation translates the adaptive governance cycle into a new ethical technique of “R&D”: Project management in *Research & Development*, the one that confines them into two parts, must shift to a more integrative practice, called here the adaptive *Reflective-Evaluative-Deliberative* cycle. These communications and knowledge systems open to a perspective bridging the biological, social, and intellectual Latourian's collective and biotic community concepts to responsibilize the former over the latter.

As shown in the case study on an antibiotic use surveillance program in animal health, being prepared means being empowered and responsible, which facilitates stakeholder engagement and even promotes collaborative nodes to accelerate changes. Preparation means joining the *community-based action*-*ethics* methodology to *R&D* practice from the start: at the time of policy (see: GPHP), program [see: (46)], and project (see: FMVUM team) ideation. Acknowledging ethics shows ways to share responsibilities among stakeholders to empower each in their respective competence for action. Empowerment ethics deepen the meaning of responsibility. Being responsible is more than accountability, it is linked to duty, proactive transparency, and scientificness as credibility and validity. Empowerment implies finding ways of acknowledging the respective position of stakeholders, notably roles, interests, missions, observations, and values, to respectfully manage multi-actor systems and share responsibility toward successful and ethical changes.

The ethical conflicts between the cognitive and the collective—as the singular will and common good—can only be managed through an open dialogue that continuously seeks *ever better* solutions, as more accuracy and consensuality. Thus, instead of questioning how to access data as a justified end^8^ to solve the antibiotic resistance problem or other One Health problems, we should look to empower the community to manage their data (a fairness *Open data* approach per community). The question we should be asking, then, is: How should we manage an *Open dialogue* between data *producers* and *users* within the community to start local changes? With empowerment ethics focusing on transparency, translation, negotiation, and arbitration, what we should call *reflexive governance*, we can engage groups and collectively drive cultural change and the willingness to accelerate it (an *Openness to data*), and then connect communities (human, animal, and ecosystem health) to reach the broader perspective of One Health and the Sustainable development of its programs, even its paradigm.

## Data Availability Statement

The original contributions presented in the study are included in the article/supplementary material, further inquiries can be directed to the corresponding author/s.

## Author Contributions

AB was responsible for the conception and ideas presented in this article, contributed to the planning, and conduct of the qualitative survey described in the article as a case study. CA and BW-J reviewed the initial manuscript, contributed to the text through changes to structure and addition of new content, and approved the final manuscript. All authors contributed to the article and approved the submitted version.

## Funding

This article was supported by funding (Ph.D. scholarships) from the Institut de Valorisation des DOnnées (IVADO), the Global One Health Network (G1HN), Centre de Recherche en Santé Public (CReSP) and the International Observatory on the Societal Impacts of AI and Digital Technologies (OBVIA). The corresponding author was an employee of the Faculté de Médecine Vétériaire at the Université de Montréal, hired to accompany the feasibility study on the implementation of a monitoring system for antibiotic use in veterinary medicine in Quebec by the Center for Expertise and Clinical Research in Animal Health and Welfare (CERCL) mandated by the Ministère de l'Agriculture, des Pêcheries et de l'Alimentation du Québec (MAPAQ).

## Conflict of Interest

The authors declare that the research was conducted in the absence of any commercial or financial relationships that could be construed as a potential conflict of interest.

## Publisher's Note

All claims expressed in this article are solely those of the authors and do not necessarily represent those of their affiliated organizations, or those of the publisher, the editors and the reviewers. Any product that may be evaluated in this article, or claim that may be made by its manufacturer, is not guaranteed or endorsed by the publisher.

## Footnotes

^1^Abduction and inquiry (a “top-down / bottom-up” approach) are key processes from pragmatism (C. S. Peirce 1839–1914 and J. Dewey 1859–1952) to bridge behaviors, norms, and beliefs, but requires ethics to function properly (8).

^2^For instance, the Stratégie québécoise de santé et de bien-être des animaux proposed an antimicrobial governance plan for all veterinary practices, the *Comité consultatif sur l'utilisation judicieuse des médicaments* for the pork industry and the professional Order as well as academic institution as initiated their own reflection. Each act on different domains (veterinary sectors), scales (professional advice up to national policies), and levels (from descriptive data to prescriptive commend or evaluative thinking).

^3^Depending on his·er background, the bioethicist can also reinforce networking, designing, applying, and synthesizing. As illustrated by the idea of “living labs” put forward by the International OBVIA, a living lab applied to bioethics can help manage power relationships, mainly those driven by interests and values (45), from an understanding of responsible conduct procedures and ethics in academic and governmental researches.

^4^See the Agri-Food AI Ateliers initiative (46) or Montréal Declaration for a Responsible Development of Artificial Intelligence deliverative processes (47).

^5^Note an ever-increasing recruitment process, involving 100 actors in 2020, to seek saturation of the perspectives, and continue the incentive process (the “interessement” according to the Latourian perspective).

^6^“Final positioning” does not exist within a collaborative governance depicted as an adaptive cycle (Step n). (Pro/post)position refers to the adaptative cycle in management (77) which must become a “constructive” and not a “disruptive” process.

^7^Economics, literally the *rules of the house* (“Okios nomos”), should join the projects in sustainable development with this general sense.

^8^For example, are the FAIR Guiding Principles a means or an end? (120). As a means, they respond to the criticisms leveled at the *Open Data movement*, when perceived as seeking “open bars.” We need the complement of a FAIRness ethic to frame responsible data sharing.
